# Draft Genome Sequence of an Erwinia tracheiphila Isolate from an Infected Muskmelon (Cucumis melo)

**DOI:** 10.1128/MRA.01058-18

**Published:** 2018-11-01

**Authors:** Lori R. Shapiro, Andres Andrade, Erin D. Scully, Jorge Rocha, Joseph N. Paulson, Roberto Kolter

**Affiliations:** a Department of Microbiology and Immunology, Harvard Medical School, Boston, Massachusetts, USA; b Stored Product Insect and Engineering Research Unit, USDA-ARS Center for Grain and Animal Health Research, Manhattan, Kansas, USA; c CIDEA Consortium, Conacyt-Centro de Investigación en Alimentación y Desarrollo, Hermosillo, Mexico; d Department of Biostatistics, Product Development, Genentech, Inc., San Francisco, California, USA; University of Maryland School of Medicine

## Abstract

Erwinia tracheiphila is a bacterial plant pathogen emerging in eastern North America. To aid in understanding genetic variation within E. tracheiphila, here we sequence the first reference genome of an infected muskmelon (Cucumis melo). The genome assembles into a single chromosomal contig, three plasmid contigs, and one bacteriophage contig.

## Abstract (precis)

Erwinia tracheiphila is a bacterial plant pathogen emerging in eastern North America. To aid in understanding genetic variation within E. tracheiphila, here we sequence the first reference genome of an infected muskmelon (Cucumis melo).

## ANNOUNCEMENT

Erwinia tracheiphila Smith (Enterobacteriaceae) is a bacterial plant pathogen that causes losses from species from only two Cucurbitaceae crop plant genera, Cucumis spp. (muskmelon and cucumber) and Cucurbita spp. (pumpkin, squash, and yellow-flowered gourds) (1, 2). E. tracheiphila is obligately insect transmitted, and it induces characteristic wilting symptoms followed by plant death within 2 to 3 weeks after symptoms first appear (3, 4). E. tracheiphila is only known to occur in temperate eastern North America, despite the worldwide occurrence of susceptible host plants (2). Recently, 88 E. tracheiphila isolates were collected and sequenced. Phylogenomic analyses found this species to be comprised of three distinct phylogenetic lineages, named *Et-melo*, *Et-C1*, and *Et-C2* (2). The majority of the E. tracheiphila isolates belong to the lineage *Et-melo,* which has the largest geographic range and infects only the introduced Cucumis sp. host plants muskmelon (Cucumis melo) and cucumber (Cucumis sativus).

Here, we report the high-quality draft genome of MDCuke, the first sequenced E. tracheiphila isolate from a muskmelon (Cucumis melo) host plant and the first draft genome sequence of an isolate from lineage *Et-melo.* MDCuke was isolated in 2009 from an infected muskmelon in Maryland.

For DNA extraction, a single colony of MDCuke was grown in liquid King’s B (KB) medium to stationary phase. Genomic DNA was extracted following the same methods for generating the reference sequence of E. tracheiphila isolate BuffGH, which was originally isolated from an infected wild gourd (Cucurbita pepo subsp. texana) and falls within the phylogenetic lineage *Et-C1* (2, 5, 6). Briefly, cells grown overnight in liquid KB medium were spun down and then lysed with 10% SDS. The lysate was treated with proteinase K, RNase, and cetyltrimethylammonium bromide (CTAB) (5) and then precipitated in ethanol. Salts, detergents, and other impurities were removed by column purification (DNA Clean & Concentrator; Zymo Research, Irvine, CA). The extracted DNA was sequenced with PacBio RS II chemistry at the University of Delaware Sequencing and Assembly Center with one single-molecule real-time (SMRT) cell, resulting in 1,916,030,430 bases sequenced, for an average coverage of 268×. The sequence was assembled using the Hierarchical Genome Assembly Process (HGAP) pipeline version 3. The genome was assembled into five contigs. The largest contig is 4,891,633 bp with 50.48% GC content, and it is likely the complete chromosome. No attempt was made to identify overlap shared by the chromosomal contig ends to circularize the genome. Three additional contigs (91,066 bp, 48,372 bp, and 39,613 bp) are putative plasmids, and one 59,759-bp contig contains all the necessary genes to be a functional bacteriophage. Coding regions were predicted by the Prokaryotic Genome Annotation Pipeline (PGAP). PGAP predicts more than 20% of the 5,116 coding sequences as pseudogenes, which is similar to the number documented in BuffGH (5). Like BuffGH, MDCuke has an abnormally high percentage of mobile DNA (6, 7). This invasion and proliferation of mobile DNA may be driving differentiation within E. tracheiphila into phylogenetically distinct groups. This first sequenced isolate from lineage *Et-melo* will help provide a basis to investigate genetic diversity within E. tracheiphila and provide data to identify possible genes underlying pathogenicity toward the different plant host genera that E. tracheiphila infects.

### Data availability.

This whole-genome shotgun project has been deposited in GenBank under the accession no. CP013970. The version described in this paper is the first version, CP013970.1. The raw reads are available under BioSample accession no. SAMN10144255 and run no. SRR7942800 in the Sequence Read Archive.
